# Is antral membrane balloon elevation truly minimally invasive technique in sinus floor elevation surgery? A systematic review

**DOI:** 10.1186/s40729-018-0123-9

**Published:** 2018-04-17

**Authors:** Huda Moutaz Asmael

**Affiliations:** Department of Oral & Maxillofacial Surgery, Dental Teaching Hospital, College of Dentistry, University of Baghdad, Bab- Almoadham, P.O.Box 1417, Baghdad, Iraq

**Keywords:** MIAMBE technique, Sinus augmentation, Sinus floor elevation surgery

## Abstract

**Background:**

Minimally invasive antral membrane balloon elevation was introduced as a less traumatic technique in sinus floor elevation surgery. This is the first systematic review to assess the results of previous studies utilizing this technique.

**Aims of the study:**

The objectives of this study were to assess the bone gain, sinus augmentation success rate, implant survival rate, and complications with minimally invasive antral membrane balloon elevation technique in comparison with the sinus floor elevation by traditional transalveolar technique (Summers’ technique).

**Materials and methods:**

An electronic search including MEDLINE (PubMed) and Cochrane database sites was conducted and supported by manual searching for articles on minimally invasive antral membrane balloon elevation from 1945 to 16 January 2017. Sometimes the researchers were contacted to fill the missing information which was not mentioned in their articles.

**Results:**

The extracted articles which involved utilization of balloon technique in maxillary sinus floor elevation surgery were 27 articles, among which only 10 articles met the inclusion criteria. The average of schneiderian membrane perforation with minimally invasive antral membrane balloon elevation (MIAMBE) technique was 6.76%. The sinus augmentation success rate ranged from 100 to 71.4% with average of 91.6%. Bone gain with this technique could reach for more than 10 mm with an average of 6.96 mm.

**Conclusions:**

Minimally invasive antral membrane balloon elevation combined the beneficial points of both lateral window approach and transalveolar approach in which it produced ≥ 10 mm of gained bone in minimally invasive manner. Anyhow, long follow-up period is needed to accurately identify the long-term success rate of dental implants placed with this technique.

## Review

Several sinus floor elevation techniques had been introduced as a minimally invasive surgical procedure. Among which, minimally invasive antral membrane balloon elevation technique was developed to achieve better results with minimal trauma to the patient also to reduce complications and intra-operative time. Conventionally, sinus augmentation procedure is performed either via lateral approach (modified Caldwell-Luc approach) [1] or through more conservative transcrestal approach (Summers’ technique) [2].

The antral membrane balloon elevation (AMBE) technique was introduced via lateral approach (direct sinus lift surgery) [3, 4].

After that, the minimally invasive antral membrane balloon elevation (MIAMBE) technique was described via transcrestal approach (indirect sinus lift) which involved utilization of balloon device through conservative 3-mm osteotomy site [5]. Since then, several articles were published utilizing this technique. This is the first systematic review for evaluation of the (MIAMBE) technique in sinus lift surgery.

### Question in focus

Is the MIAMBE effective in the terms of sinus augmentation success rate, survival rate of dental implants, bone gain, and complication rate compared with the conventional sinus floor elevation by transalveolar technique (Summers’ technique)?

## Materials and methods

### Search strategies

This study was executed following the PRISMA criteria for the systematic review. An electronic search including MEDLINE (PubMed) and Cochrane database sites was conducted and supported by manual searching for targeted articles through the related journals and web sites from 1945 to 16 January 2017.

### Inclusion criteria

1. Prospective, retrospective studies and randomized clinical trials.
2. Articles published in English language only.
3. Human studies.
4. Healthy patients without systemic or local disease that may affect the maxillary sinus health or the sinus lift procedure outcome.
5. Studies which included at least six patients.
6. Sinus floor elevation via the transcrestal approach only (indirect sinus lift).
7. Follow-up period of at least 6 months.

### Exclusion criteria

1. Case reports and studies which included less than six patients.
2. Studies published in other language than English.
3. Experimental (animal studies).
4. Sinus floor elevation via lateral approach.
5. Maxillary sinus pathology or presence of sinus septa.
6. Studies with follow up period of less than 6 months.

### The process of extracting articles

The following keywords were involved in the electronic search:

MAILLARY SINUS AUMENATION, SINUS LIFT, INDIRECT SINUS LIFT, ANTRAL MEMEMBRANE ELEVATION, MINIMALLY INVASIVE ANTRAL BALLOON ELEVATION, ATROPHIC MAXILLA, SINUS FLOOR ELEVATION, SINUS MEMBRANE ELEVATION

**Fig. 1:**
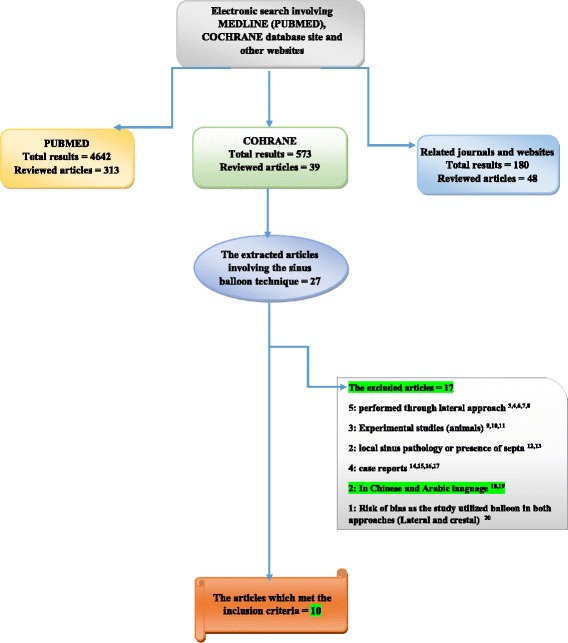
Flow chart showing the search strategy

The results (abstracts and articles) were reviewed twice by the same author at different time intervals. Hand searching for the full-text articles bibliographies of the selected studies was established. Sometimes the researchers were contacted to fill the missing information which was not mentioned in their articles or for more explanation about their results. The search process was demonstrated in (Fig. 1).

## Results

The total electronic search results were 5395 articles. The reviewed articles were 400, and the extracted articles which involved utilization of balloon technique in the maxillary sinus floor elevation surgery were 27 articles. Siventen articles were excluded from this study [6–20] and only 10 articles met the inclusion criteria.

The results of the selected studies were categorized to assess the success rate of sinus augmentation by MIAMBE technique, to report the perforation rate of schneiderian membrane and to identify the rate of complications associated with MIAMBE technique as shown in Table 1. The survival rate and failure rates of dental implants placed in the augmented maxillary sinus were calculated for the selected studies and registered in Table 2. The average of schneiderian membrane perforation was calculated for the ten extracted studies utilizing MIAMBE technique, and it was 6.76%. The sinus augmentation success rate reported ranged from 100 to 71.4% with an average of 91.6%. The average of implant survival rate in these studies was 96.62%. Regarding the grafting material, synthetic bone graft was utilized in five studies. Four studies used a mixture of autogenous and synthetic bone graft while one study utilized allogeneic cancellous bone graft. PRF (platelets rich fibrin) mixed with either autogenous or synthetic bone graft was used in the five studies.

**Table 1:** Descriptive statistics demonstrate patient characteristics and outcomes of MIAMBE technique in the selected studies

Study	Patients		Age (years)	*N* of sinus augmentation	Sinus augmentation success rate %	Baseline bone height (mm)	Bone gain (mm)		Antral membrane elevation (mm)	Total bone height (mm)	Inflate balloon volume	Type of graft	Membrane perforation		Test for membrane perforation	Complications	Follow-up period after operation
	N	M:F					R	M					N	PR %			
Kfir et al. [5]	12	NM	42 ± 9	NM	91.6	3.7 ± 1.4	NM		> 10	10–17	≤ 2.5 mL	-PRF -ABP -Bi-Ostetic synthetic bone graft	1	8.33	Valsalva maneuver Direct visualization	1 membrane tear 1 balloon rupture 1 implant failed	23 months
	12	NM		NM	100	3.5 ± 1.3	NM		> 10	10–18			0	0		1 mild periprocedural nosebleed	12 months
Kfir et al. [29]	36	M = 18 F: 18	42 ± 9	36	97.2	3.4 ± 2.1	NM		> 10	8–18	NM	-PRF -ABP -Bi-Ostetic Synthetic bone graft -Fisiograft gel	1	2.77	Valsalva maneuver	1 membrane tear 2 implant failed	6–8 months
Hu et al. [30]	28	M = 143 F = 14	40.2 ± 12.35	28	92.85	4.92 ± 1.24	NM		Mean 10.9 ± 2.06	NM	0.67 ± 0.17 mL	-PRF -Bio-Oss	2	7.14	Valsalva maneuver	1 mild nosebleed after surgery 2 membrane tear and the cases aborted	15.9 ± 2.94 months.
Kfir et al. [31]	112	M = 50 F = 62	44.1 ± 12.9	NM	97.3% initial procedural success 100% secondary procedural success	3.8 ± 2.1	NM		> 10	11–18	NM	-PRF -Synthetic bone graft -ABP -Fisiograft gel	12	10.71	Valsalva maneuver Direct visualization	1 infection and oroantral fistula at 4 weeks 3 membrane tear and the procedures aborted **9** micropuncture	13 months
Mazor et al. [32]	20	NM	37–72	24	100	2–6	NM		11	NM	NM	-PRF -Synthetic bone substitute	0	0	Valsalva maneuver Direct visualization	1 patient was allergic to the antibiotic	18 months
Petruzzi et al. [33]	40	M = 16 F = 24	41.5	NM	NM	8.00 ± 2.19		0.6	≤ 20	14.66 ± 1.48	1– ml	Calcium sulphate solution	3	7.5	Microscope (KarlKaps)	1 m acrolaceration 2 m icrolacerations 4 hemifacial edema	1 year
Peñarrocha-Diago et al. [34]	6	M = 5	27–51	6	83.3% for the all 6 cases 100% for the five cases	2.1–4.1	7.2–10.8	8.7	NM	11.3–14.5	NM	-ABP -BIO-OSS bovine bone grafts	1	16.66	(Medi Pack Pal) endoscope	1 membrane tear and case aborted	1 year after prosthetic loading
Gonzalez et al. [35]	14	M = 7 F = 7	NM	NM	71.4	5.2		8.5	NM	NM	NM	BIO-OSS bovine bone grafts	3	21.32	An operating microscope	Failure of four cases due to mucosa perforation (21.32%) and balloon breakage (7.14%). 1 implant showed marginal periimplantitis which treated successfully	1 year
Dhandapani et al. [36]	9	NM	25–60	10	100	5–8	3–5.5	4.34	≥ 10	13.5–9	1 cc	Irradiated allogeneic cancellous bone and marrow graft	0	0	NM	No complications	6 months
Asmael, and Lateef [37]	13	M = 4 F:9	28–57	17	100	2.3–7.8	4.9 –10.6	6.70	NM	9.8–17.2	0.5–1 cc	Particulate bone grafts (βTricalcium Phosphate)	0	0	Direct vi ion Hydraulic pressure	1 mild nasal bleeding 1 infraorbital ecchymosis	1 year
Total : Average Total : Range					91.6 100–71.4%		6.968 3–10.8 mm						6.76 0–21.32%				

*MIAMBE* minimally invasive antral membrane balloon elevation, *N* number, *NM* not mentioned, *M:F* male: female numbers, *M* mean, *R* range, *PRF* platelets rich fibrin, *PRP* platelets rich plasma, *ABP* autogenous bone particles, *PR* perforation rate

**Table 2:** Summary of the dental implants characteristics, survival rates, and failure rates with MIAMBE technique

Study	*N* of patients	Baseline bone height	Total *N* of implants	Implants survival rate %	*N* of failed implants	Implant failure rate %	Implant lengths	Implant diameters	Timing of failure	Follow-up period after operation
Kfir et al. [5]	12	3.7 ± 1.4 mm	Mean ± SD 2.08 ± 0.51	NM	1	NM	13, 17.1 mm	3.75, 5 mm	2 weeks after procedure	23 months
	12	3.5 ± 1.3 mm Mean = 3.5	Mean ± SD 1.91 ± 0.51	100	0	0	13, 17.1 mm	3.75, 5 mm		12 months
Kfir et al. [29]	36	3.4 ± 2.1 mm	72	97.2	2	2.77	13, 17.1 mm	3.75, 5 mm	1 and 3 weeks after procedure	6–8 months
Hu et al. [30]	28	4.92 ± 1.24 mm	26	96.15	1	3.84	NM	3.8, 5.0 mm	2 weeks after procedure	15.9 ± 2.94 months.
Kfir et al. [31]	112	3.8 ± 2.1 mm	219	95	11 FR = 5%	5	13, 17.1 mm	3.75, 5 mm	At 6 months after procedure	13 months
Mazor et al. [32]	20	2–6 mm	37	100	0	0	13 mm	5 mm		18 months
Petruzzi et al. [33]	40	8.00 ± 2.19 mm	56	100	0	0	11.5, 13, and 15 mm	4.00, 6.50 mm		1 year
Peñarrocha-Diago et al. [34]	6	2.1–4.1 mm	6	100	0	0	10, 11.5 mm	4.2, 5.2 mm		1 year (after prosthetic loading)
Gonzalez et al.[35]	14	5.2 mm	11	90	1	10	13 mm	NM	1 year	1 year
Dhandapani et al. [36]	9	5–8 mm	NM	NM	NM	NM	NM	NM	NM	6 months
Asmael and Lateef [37].	13	2.3–7.8 mm	23	91.30	2	8.70	10, 12 mm	4.2, 4.3, 4.8, and 5 mm	At 1 and 6 months	1 year

*MIAMBE* minimally invasive antral membrane balloon elevation, *N* number, *NM* not mentioned

## Discussion

Sinus floor elevation surgery with balloon is said to be a minimally invasive technique [5]_,_ but to date, no systematic review was made to clearly present the study results, authors experience, and surgical outcomes. Results of studies that utilized MIAMBE technique could be discussed under these highlighted points.

### Maxillary sinus entry and elevation of sinus membrane

There are two critical points in sinus floor elevation surgery which include entry to the sinus and elevation of schneiderian membrane. Several atraumatic techniques had been developed to make transalveolar approach more predictable among which minimally invasive methods introduced like MIMBE technique [5], novel drills, and reamers to aid in atraumatic entry to the sinus [21]. Also, the Jeder-System which utilize hydraulic pressure had been introduced with predictable results [22]. Anyhow, the outcomes of these techniques need to be compared to reach to a reliable clue about the most effective method in sinus lift surgery.

### Sinus augmentation and bone gain

The success of sinus augmentation procedure with MIAMBE technique was ranged from 100 to 71.4% with an average of 91.6% in these studies. Bone gain with MIAMBE technique could reach for more than 10 mm, it ranged from 3 to 10.8 mm with an average of 6.96 mm. It should be mentioned that some articles failed to report the gained bone in details.

The traditional procedure (Summers’ technique) had a limitation of allowing for only a minimal amount of bone gain which is 3–4 mm. While sinus floor elevation surgery via lateral approach produced a huge elevation ≥ 10 mm [23], it is considered as an invasive technique.

### Implants survival rates

Implant survival rate associated with MIAMBE technique was ranged from 90 to 100% with an average of 96.62% as shown in Table 2. On the other hand, systematic reviews have evaluated the implant survival rate after osteotome-mediated sinus floor elevation surgery which shows an implant survival rate higher than 90% [24–26]. In most of MIAMBE studies, dental implant failure occurred early during the first 6 months after operation, some authors mentioned the cause for implant failure which was associated with infection, and others did not addressed the cause.

### Surgical complications

#### Intra-operative complications

The most common intra-operative complication associated with sinus lift procedure was sinus membrane tear [27]. The rate of schneiderian membrane perforation with MIAMBE technique was ranged from 0 to 21.32% with an average of 6.76%. This rate was similar to the schneiderian membrane perforation rate (0–21.4%) which was reported in the systematic review of sinus floor elevation success via transalveolar approach by Tan [28].

In some of these studies, membrane perforation was treated successfully with collagen membrane and the procedure continued with successful MIAMBE technique; other studies aborted the procedure. Furthermore, some authors demonstrated the causes of sinus membrane perforation which could be due to the too rapidly inflated balloon, balloon rapture, and fracture of the sinus floor during the osteotome procedures. Anyhow, with minimally invasive methods, the accuracy in the diagnosis of sinus membrane perforation is difficult without the availability of endoscope. Therefore, the perforation rates in these studies should be interpreted carefully, and the tests utilized to detect the perforation should be addressed accurately Table 1. An important point to the surgeons who executed this procedure is to check for membrane integrity after each surgical step by endoscope, Valsalva maneuver, direct vision, and/or by aspiration with normal saline to accurately report the cause of perforation.

#### Post-operative complications

Complications registered with MIAMBE technique in these studies involved sinus membrane perforation, implant failure, infection, oroantral fistula, balloon rapture, mild self-limiting nose bleeding, and infra-orbital ecchymosis. All studies reported less post-operative pain, bleeding, and discomfort on the patient side. On the surgeon side, it offered short learning curve and less surgical time.

This systematic review detected several shortcomings in the studies utilized (MIAMBE technique), these include:

- One study was not critical in the presentation of its results and did not include the failed aborted cases in the total sinus augmentation success rate.
- Some studies failed to report the number of sinus augmentation procedures as it differed from the number of the patients enrolled in these studies.
- Some did not mention the cause of membrane perforation or implant failure.
- Some studies did not mentioned well-defined implant survival or success criteria according to which they depend in reporting the survival rate of implants.
- Lack of long follow-up period in most of these studies.
- Lack of randomized clinical trial (RCT) studies as shown in (Table 1).

## Conclusion

MIAMBE technique is proved to be a minimally invasive procedure which is associated with low post-operative complications. The amount of gained bone with MIAMBE technique is predictable and comparable with the amount of bone achieved with the more invasive lateral window technique. Anyhow, long follow-up period is needed to accurately identify the long-term success rate of dental implants placed with this technique.

## Abbreviations

ABP: Autogenous bone particles
AMBE: Antral membrane balloon elevation
M: Mean
M:F: Male:female numbers
MIAMBE: Minimally invasive antral membrane balloon elevation
N: Number
NM: Not mentioned
PR: Perforation rate
PRF: Platelets rich fibrin
PRP: Platelets rich plasma
R: Range
RCT: Randomized clinical trial
